# Antisense oligonucleotides to class III β-tubulin sensitize drug-resistant cells to Taxol

**DOI:** 10.1038/sj.bjc.6690507

**Published:** 1999-06

**Authors:** M Kavallaris, C A Burkhart, S B Horwitz

**Affiliations:** Department of Molecular Pharmacology, Albert Einstein College of Medicine, Bronx, New York 10461, USA; Children's Cancer Research Institute, Sydney Children's Hospital, Randwick, New South Wales 2031, Australia

**Keywords:** Taxol, antisense, tubulin, lung cancer, drug resistance

## Abstract

A major impediment to the successful use of Taxol in the treatment of cancer is the development of drug resistance. The major cellular target of Taxol is the microtubule that is comprised of α- and β-tubulin heterodimers. Binding sites for Taxol have been delineated on the β-tubulin subunit that has six isotypes. We have recently described increased expression of the brain-specific human class III β-tubulin isotype, encoded by the Hβ4 gene, in both Taxol-resistant ovarian tumours and non-small-cell lung cancer cell lines. To evaluate directly the role of the class III β-tubulin isotype in mediating Taxol resistance, antisense phosphorothioate oligodeoxynucleotides (ODN) targeted against various regions of the Hβ4 gene have been designed and examined for their efficacy in reducing Hβ4 gene and protein expression. Taxol-resistant lung cancer cells, A549-T24, which are 17-fold resistant to Taxol and display a fourfold increase in Hβ4 expression compared to the parental A549 cells, were treated with 1 μM antisense ODNs. Two ODNs, AS1 and AS3, were found to reduce mRNA expression by 40–50%, as determined by reverse transcription polymerase chain reaction. A concentration-dependent reduction in Hβ4 mRNA expression was demonstrated with AS1 ODN. Immunofluorescence staining of cells treated with AS1 ODN revealed a decrease in class III protein expression which corresponded to a 39% increase in sensitivity to Taxol (*P* < 0.005). These findings support an important role for Hβ4 (class III) β-tubulin expression in Taxol resistance and have potential implications for the treatment of Taxol-resistant tumours. © 1999 Cancer Research Campaign


*British Journal of Cancer* (1999) **80**(7), 1020–1025
© 1999 Cancer Research Campaign
Article no. bjoc.1999.0457

### Antisense oligonucleotides to class III β-tubulin sensitize drug-resistant cells to Taxol

**M Kavallaris[1,2], CA Burkhart[1] and SB Horwitz[1]**

[1]Department of Molecular Pharmacology, Albert Einstein College of Medicine, Bronx, New York 10461, USA; [2]Children's Cancer Research Institute, Sydney Children's Hospital, Randwick, New South Wales 2031, Australia

**Summary** A major impediment to the successful use of Taxol in the treatment of cancer is the development of drug resistance. The major cellular target of Taxol is the microtubule that is comprised of α- and β-tubulin heterodimers. Binding sites for Taxol have been delineated on the β-tubulin subunit that has six isotypes. We have recently described increased expression of the brain-specific human class III β-tubulin isotype, encoded by the Hβ4 gene, in both Taxol-resistant ovarian tumours and non-small-cell lung cancer cell lines. To evaluate directly the role of the class III β-tubulin isotype in mediating Taxol resistance, antisense phosphorothioate oligodeoxynucleotides (ODN) targeted against various regions of the Hβ4 gene have been designed and examined for their efficacy in reducing Hβ4 gene and protein expression. Taxol-resistant lung cancer cells, A549-T24, which are 17-fold resistant to Taxol and display a fourfold increase in Hβ4 expression compared to the parental A549 cells, were treated with 1 μM antisense ODNs. Two ODNs, AS1 and AS3, were found to reduce mRNA expression by 40–50%, as determined by reverse transcription polymerase chain reaction. A concentration-dependent reduction in Hβ4 mRNA expression was demonstrated with AS1 ODN. Immunofluorescence staining of cells treated with AS1 ODN revealed a decrease in class III protein expression which corresponded to a 39% increase in sensitivity to Taxol ($P < 0.005$). These findings support an important role for Hβ4 (class III) β-tubulin expression in Taxol resistance and have potential implications for the treatment of Taxol-resistant tumours.

**Keywords:** Taxol; antisense; tubulin; lung cancer; drug resistance

Taxol is an important anti-tumour agent that is particularly effective in the treatment of ovarian and breast carcinomas, although its usefulness is often hampered by the development of drug resistance. Taxol binds to the β-tubulin subunit of the microtubule (Rao et al, 1995) of which six distinct β-tubulin isotypes have been identified in mammalian cells (Lewis and Cowan, 1990; Luduena, 1998). Tubulin forms a heterodimer that consists of α- and β-tubulin subunits that constitute the microtubule. Microtubules are an integral part of the cytoskeleton and play a crucial role in chromosomal separation and segregation (Hyams and Lloyd, 1994). The genes that encode tubulin have been highly conserved throughout evolution and, even within species, multiple α- and β-tubulin genes encode distinct tubulin gene products (Sullivan & Cleveland, 1986).

Taxol resistance has been attributed to a number of mechanisms including induction of the multidrug resistance phenotype and overexpression of P-glycoprotein (Horwitz et al, 1993). In addition, changes in the composition and mutations in β-tubulin isotypes have been identified in cells resistant to Taxol (Haber et al, 1995; Jaffrezou et al, 1995; Dumontet et al, 1996; Giannakakou et al, 1997; Kavallaris et al, 1997; Ranganathan et al, 1998*a*). We recently reported that expression of specific β-tubulin isotypes increased in Taxol-resistant epithelial ovarian tumours and that these changes were similar to those identified in Taxol-resistant human lung cancer cells, A549-T12 and A549-T24 (9- and 17-fold resistant to Taxol respectively) (Kavallaris et al, 1997). The Taxol-resistant ovarian tumours displayed significant increases in two brain-specific β-tubulin isotypes, Hβ4 (class III) and H5β (class IVa), which are not normally expressed in epithelial tissues.

Microtubule dynamics can be influenced by the composition of β-tubulin isotypes, and aberrant expression of specific isotypes could confer a survival advantage to cells when exposed to a drug such as Taxol. There is evidence that altering the β-tubulin isotype composition in vitro can modulate microtubule dynamics in response to Taxol (Derry et al, 1997). Microtubules composed of class III (Hβ4 gene product), or class IV (H5β and Hβ2 gene products) β-tubulin were significantly less sensitive to the effects of bound Taxol than microtubules assembled from unfractionated tubulin. This finding strongly suggests that alterations in β-tubulin isotype composition may play a role in resistance to antimitotic agents that target the tubulin/microtubule system. While there is increasing support for the idea that β-tubulin isotype composition alters microtubule dynamics and response to drugs such as Taxol in vitro (Banerjee et al, 1990; Lu and Luduena, 1993; Derry et al, 1997), there is limited knowledge concerning its role in Taxol resistance in cells. Recently, Ranganathan et al (1998*b*) demonstrated that in human brain cell lines intrinsically expressing different levels of class III β-tubulin, those cell lines with the higher level of this isotype were less sensitive to the effects of Taxol. To date, there is no direct evidence that altered expression of the brain-specific class III β-tubulin isotype in Taxol-resistant cells contributes to the resistance phenotype.

Antisense oligonucleotides have been used successfully to block the expression of a number of genes so that their function and cellular interactions could be better elucidated (Helene and Toulme, 1990). To identify a possible role for an increased expression of β-tubulin isotype, Hβ4, in Taxol resistance, antisense oligodeoxynucleotides (ODNs) were targeted to various regions of

*Received 22 September 1998*
*Revised 20 January 1999*
*Accepted 26 January 1999*

*Correspondence to:* SB Horwitz

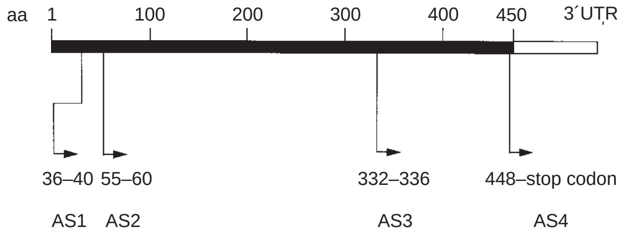

**Figure 1** Schematic diagram of Hβ4 cDNA sequence. Sequences were selected against Hβ4 β-tubulin in regions where the greatest divergence between the isotypes exist. The location of the antisense sequence is listed by amino acid position and the name of each antisense oligonucleotide is listed below each indicated sequence (Genbank accession U47634)

**Table 1** Oligonucleotide sequences

| Oligomer | Sequence[a] | Target |
|---|---|---|
| AS1 | ACGTGGGCGACTCGG | ORF[b] |
| AS2 | TCTTCTCACAAGTACGTG | ORF |
| AS3 | TTGCTCTGGATGGCC | ORF |
| AS4 | GCGCCAAGTGAAACTG | ORF and stop codon |
| SCR 1 | CTTACTAGCTTAGTACGA | Scrambled ODN |
| SCR 2 | GCCGCAGTCGGAGTG | Scrambled ODN |

[a]All sequence entries written in the 5′ to 3′ direction. [b]ORF, open reading frame.

the Hβ4 gene. The effect of antisense oligonucleotide treatment on Taxol-resistant A549-T24 cells was characterized at the mRNA and protein levels. Antisense oligonucleotides successfully decreased the expression of the class III β-tubulin isotype in Taxol-resistant non-small-cell lung cancer cells. Decreased expression of the class III β-tubulin isotype corresponded with an increased sensitivity to Taxol. This is the first report implicating a direct relationship between Taxol resistance and altered expression of a brain-specific isotype in tumour cells of epithelial origin.

### MATERIALS AND METHODS

### Cell culture and treatment with oligonucleotides

Human non-small-cell lung cancer cells (A549-T24) selected for resistance to Taxol, were maintained in 24 nM Taxol, RPMI-1640 containing 1% penicillin–streptomycin (Gibco Laboratories, Grand Island, NY, USA) and 10% fetal bovine serum (FBS) (Kavallaris et al, 1997). Phosphorothioate ODNs designed to various regions of the Hβ4 gene (Figure 1) were synthesized at Gene Link (Thornwood, NY, USA) as shown in Table 1. Lipofectin was preincubated in OPTI-MEM (20 μg $ml^{-1}$ Lipofectin; Gibco Laboratories, Grand Island, NY, USA) for 40 min at room temperature prior to the addition of various concentrations (as specified in the figure legends) of phosphorothioate ODN in OPTI-MEM for 15 min at room temperature to allow for the formation of ODN–lipofectin complexes. A549-T24 cells were plated onto 35-mm dishes in 2 ml RPMI-1640 containing 4 nM Taxol and 10% FBS at a concentration that achieved approximately 60% confluency within 24 h. Cells were washed twice with 1 ml OPTI-MEM, followed by the addition of ODN–lipofectin complexes and further incubated at 37°C for 4 h. Cells were washed and incubated in fresh RPMI medium at 37°C for 20 h. After a final round of washing, the cells were treated using the same procedure with half the concentration of ODN (0.5 μM) unless otherwise stated. A549-T24 cells were maintained in 4 nM Taxol throughout the duration of each experiment. This concentration was chosen so as to avoid increased Taxol toxicity in cells exhibiting down-regulation of the class III β-tubulin isotype. Conditions were selected so that there was less than 10% difference in the number of viable cells in the ODN-treated versus lipofectin only samples, as determined with a vital dye assay. All experiments included the use of a scrambled ODN and lipofectin only control.

### Cellular accummulation and intracellular distribution of FITC-labelled oligonucleotide

The accumulation of fluorescein isothiocyanate (FITC)-labelled (Gibco Laboratories) ODNs in cells was monitored using a fluorescence microscope. FITC-ODN had the same base composition and length as AS1 ODN. A549-T24 cells were plated on coverslips in 35-mm dishes, incubated at 37°C for 24 h and treated once with 1 μM FITC-labelled ODN prior to monitoring cellular uptake. Briefly, cells were harvested at 4 and 24 h following ODN treatment, fixed with 70% ethanol–phosphate-buffered saline (PBS) for 20 min and mounted on glass slides using 70% glycerol in PBS. Cells were visualized for uptake and intracellular distribution of FITC-labelled oligonucleotides with a Zeiss Axioskop microscope fitted with epifluorescence illumination.

### Determination of β-tubulin gene expression by RT-PCR

Following ODN treatment of the A549-T24 cells, β-tubulin analysis was performed as previously described (Kavallaris et al, 1997). Briefly, total cellular RNA was isolated from ODN treated A549-T24 cells, DNase I treated to remove any contaminating DNA, and reverse transcribed for reverse transcription polymerase chain reaction (RT-PCR) analysis. Experiments were performed twice, with three independent PCR reactions being done for each sample.

### Immunofluorescence and digital scanning

A549-T24 cells were grown on glass coverslips to approximately 60% confluency. The cells were treated as described under ‘Cell culture and treatment with oligonucleotides’. Briefly, cells were subjected to two ODN (1 and 0.5 μM respectively) treatments which were 24 h apart, prior to staining the cells with either monoclonal antibody (mAb) to α-tubulin (Sigma; 1:200 dilution, 1% bovine serum albumin (BSA) in PBS), or mAb to class III β-tubulin (Sigma; 1:50 dilution, 1% BSA in PBS) and detection with

© 1999 Cancer Research Campaign

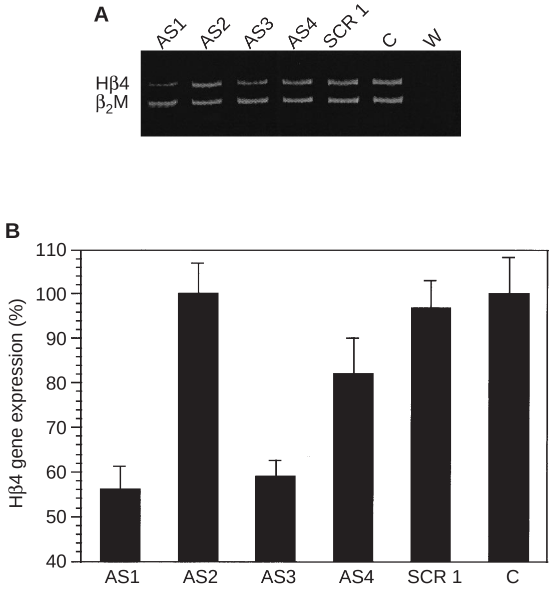

**Figure 2** Effect of antisense oligonucleotides on Hβ4 β-tubulin expression. A549-T24 cells were treated with 1 μM ODN as described in Materials and Methods. RNA was isolated 24 h after the second ODN treatment and reverse transcribed. Competitive RT-PCR analysis of Hβ4 β-tubulin gene expression using $\beta_2$-microglobulin as an internal standard was performed. (**A**) Representative gel of the Hβ4 β-tubulin and $\beta_2$-microglobulin PCR products from A549-T24 cells following treatment with ODNs. RT-PCR products were separated on 10% PAGE followed by ethidium bromide staining. Lane C, lipofectin only control; Lane W, water control. (**B**) Ratios between the Hβ4 PCR product and the $\beta_2$-microglobulin PCR product were determined and results were expressed as a percentage of the mean value of the lipofectin only control. The experiment was done twice and the results represent three independent RT-PCR reactions for each experiment

Cy3-conjugated anti-mouse IgG (Amersham 1:1000, 1% BSA in PBS). Fluorescence was quantitated using an Olympus IX70 inverted microscope and digitizing the images with a Photometrics PXL camera as previously described (Kavallaris et al, 1997).

### Growth inhibition assays

Cells were plated in triplicate at a concentration of $1 \times 10^5$ cells per ml (2 ml per well) in 6-well plates and allowed to attach for 24 h prior to treatment with ODN as described for the immunofluorescence procedure. Following the two ODN treatments (1 and 0.5 μM respectively), A549-T24 cells were exposed to varying amounts of Taxol (0–20 nM) for 72 h. The half-life of phosphorothioate ODNs is in excess of 48 h (Wagner, 1994); therefore, the cells were treated twice to ensure that optimum down-regulation of class III β-tubulin was maintained during the cytotoxicity assay. Cells were trypsinized and counted on a Coulter counter (model ZF0031; Coulter Electronics Inc., Hialeah, FL, USA). Three independent experiments using triplicate plates were performed.

### RESULTS

### Effect of oligonucleotides on Hβ4 mRNA levels

Antisense oligodeoxynucleotides (ODN) have been designed for complimentarity to various regions of the human β-tubulin Hβ4 gene (Figure 1 and Table 1). Due to the high nucleotide homology of the six human β-tubulin isotypes, limited regions were available on the Hβ4 gene to design antisense ODNs with high specificity and no cross-reactivity. Four ODNs were synthesized as well as a general scrambled ODN control, SCR 1 (Table 1). In initial experiments, cells were treated with 1 μM ODN for 4 h. No difference in cell membrane integrity was found between the antisense-treated and control cells as determined by the trypan blue exclusion dye over a 72-h period (data not shown). Hβ4 gene expression was determined by competitive RT-PCR (Kavallaris et al, 1997). Following two treatments with 1 μM ODN, 24 h apart, two of the antisense ODNs, AS1 and AS3, were found to decrease the expression of the Hβ4 gene by approximately 40–50% (Figure 2). These results were obtained in two separate experiments, with the PCR being performed in triplicate for each run. Subsequent experiments utilized AS1 ODN and its corresponding control, SCR 2 ODN, as AS1 ODN was able to substantially decrease the Hβ4 message within 24 h from the initial ODN exposure, while AS3 ODN required two treatments (data not shown).

### Uptake and cellular distribution of ODN

To ensure that the antisense ODN effect was due to efficient accumulation of the ODN–lipofectin complex, A549-T24 cells were examined for their ability to accumulate FITC-AS1 ODN (Figure 3). Following a 4-h treatment with FITC-AS1 ODN, all of the cells showed substantial cytoplasmic localization in granular compartments and nuclear accumulation. A similar effect was noted when cells were observed at 24 h. The accumulation of fluorescence in the cells is likely to represent FITC-ODN, and not disassociated FITC, due to the stability of phosphorothioate ODNs in culture (Akhtar and Juliano, 1992; Wagner, 1994).

### Concentration-dependent reduction of Hβ4 message by AS1 ODN

Treatment of A549-T24 cells with AS1 ODN resulted in a concentration-dependent inhibition of Hβ4 message (Figure 4). Reduction of Hβ4 message levels was observed at concentrations of 0.1 μM AS1 ODN. At 1 μM, maximum specific effects were observed following treatment, which resulted in a 46% decrease in message compared to 1 μM of the matched scrambled control, SCR 2 ODN. No concentration-dependent specific effect on Hβ4 message levels was observed with the SCR 2 ODN and there was no significant difference between the SCR 2 ODN and the lipofectin only control (Figure 4). Higher levels of AS1 were tested but resulted in unacceptable levels of toxicity (> 10% cell death; data not shown). Gene expression of the constitutively expressed β-tubulin isotype HM40 (class I) in the A549-T24 cells was unaltered following AS1 treatment compared to SCR 2 and lipofectin only control (Figure 5). Expression levels of the β-tubulin isotype Hβ9 (class II) gene in the A549-T24 cells were unchanged between AS1 and SCR 2, although these levels were slightly higher than those observed in the lipofectin only control (Figure 5). The levels of the other three β-tubulin isotypes, H5β, Hβ2 and Hβ1, were not altered following AS1 treatment (data not shown). In contrast, there was a decreased expression of the class III β-tubulin isotype, Hβ4, compared to either the SCR 2 or lipofectin only control.

### Inhibition of class III β-tubulin protein

To establish whether a decrease in Hβ4 gene product corresponded to a decrease in the class III β-tubulin protein, antisense-treated

 © 1999 Cancer Research Campaign

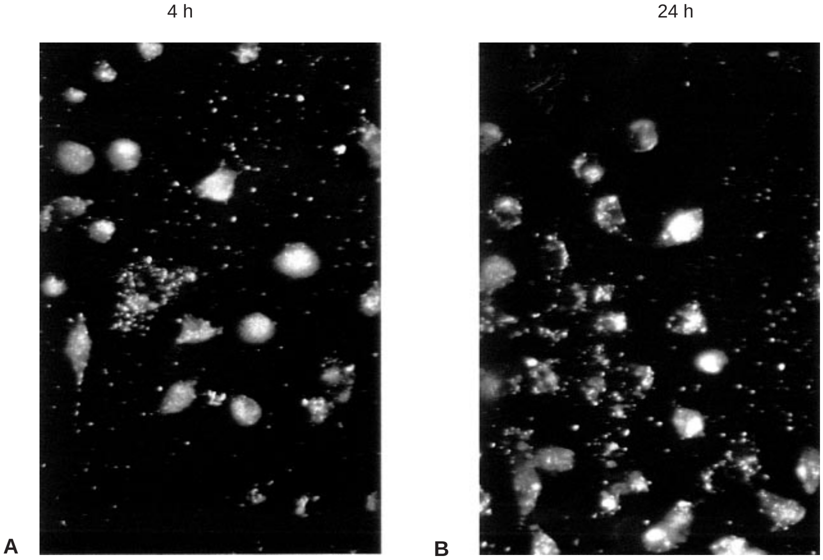

**Figure 3** Incorporation and distribution of FITC-AS ODN in A549-T24 cells. Cells were treated with 1 μM FITC-AS1 and examined at 4 (**A**) and 24 h (**B**) after initial treatment as described in Methods. Cells were imaged on a fluorescence microscope using a 60× oil immersion objective

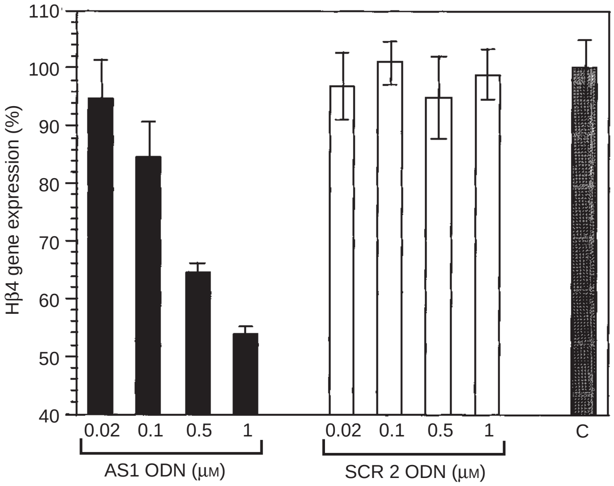

**Figure 4** Dose–response for oligonucleotide inhibition of Hβ4 message. A549-T24 cells were treated with 0.02–1 μM AS1 (solid bar) or SCR 2 (white bar) for 4 h and expression analysis was performed as described for Figure 2. C, Lipofectin only control (grey bar)

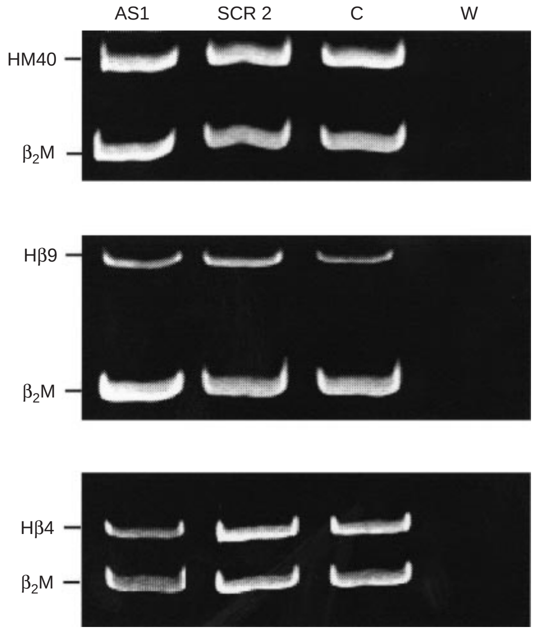

**Figure 5** RT-PCR determination of β-tubulin isotypes following AS treatment. A549-T24 cells were treated with 1 μM AS1 or SCR 2 (matched scrambled ODN) for 4 h and expression analysis was performed as described for Figure 2. Competitive RT-PCR, involving coamplification of either HM40, Hβ9 or Hβ4, and control $\beta_2$-microglobulin gene sequences, were subjected to 35 cycles and the products were separated on 10% PAGE followed by ethidium bromide staining. Lane W, water control

A549-T24 cells were stained with mAb to either α-tubulin or class III β-tubulin and visualized with a fluorescent secondary antibody.

Immunostained cells were imaged with a digital camera attached to a fluorescence microscope and the mean fluorescence intensity of at least 100 individual cells was measured after AS1 ODN, SCR 2 ODN or lipofectin only treatments (Figure 6). This technique has previously been used to measure tubulin levels in cells (Kavallaris et al, 1997). A clear decrease in class III β-tubulin immunofluorescence intensity was observed in the antisense-treated cells compared to both the SCR 2 and lipofectin only controls. In contrast, the fluorescence intensity of total α-tubulin expression in AS1-treated cells gave a similar frequency distribution to SCR 2 and lipofectin only control.

### Effect of antisense ODN on Taxol sensitivity

After establishing that AS1 ODN could decrease Hβ4 expression at both the message and protein levels, 72 h cytotoxicity assays were performed on treated cells to determine whether this decrease resulted in modulation of Taxol resistance in A549-T24 cells. Cytotoxicity assays demonstrated a significant increase of 39.3% in Taxol sensitivity in the A549-T24 cells treated with AS1 compared

*© 1999 Cancer Research Campaign*

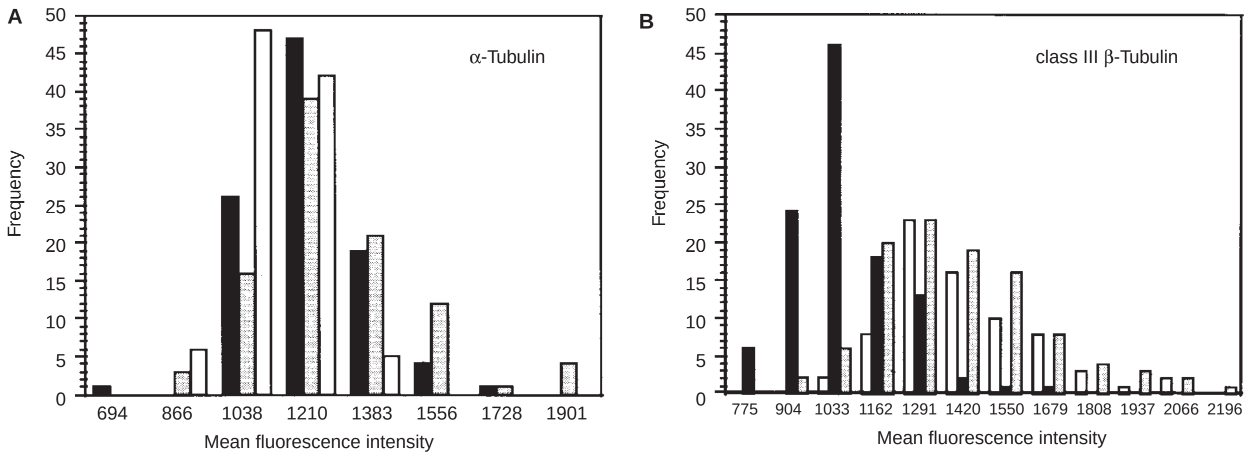

**Figure 6** Distribution of fluorescence intensity as a measurement for tubulin expression in A549 cells treated with antisense oligonucleotides. A549-T24 cells were treated twice with either AS1, SCR 2 (1 and 0.5 μM), or lipofectin only control for 4 h, with an interval of 24 h between initial exposures. Cells were probed with mAb to either total α-tubulin (**A**) or class III β-tubulin (**B**) 24 h after the second treatment. The average pixel intensity was determined for a minimum of 100 stained cells. Intensity distribution was determined for each treatment. Treatments were as follows: black bars, AS1; grey bars, SCR 2; and white bars, lipofectin only control. This experiment was done three times with similar results being obtained each time. This immunofluorescence data is a representative experiment

**Table 2** Effect of β-tubulin Hβ4 antisense ODN treatment on Taxol sensitivity in A549-T24 cells

| ODN treatment | $ID_{50}$[a] (nM Taxol) | Increase in Taxol sensitivity (%)[b] |
|---|---|---|
| AS1 | 46.6 ± 3.3 | 39.3[c] |
| SCR2 | 76.7 ± 5.5 | – |
| Control | 61.2 ± 5.3 | – |

[a]$ID_{50}$, drug concentration that inhibits cell division by 50% after 72 h.
[b]Determined by calculating the % change between AS1 and SCR2.
[c]$P < 0.005$.

to the A549-T24 cells treated with the scrambled ODN ($P < 0.005$) (Table 2). A lipofectin only control was included in each experiment and a significant increase in Taxol sensitivity was also observed in the AS1 ODN-treated cells compared to this control ($P < 0.05$). Three independent experiments were performed.

### DISCUSSION

Taxol is an effective chemotherapeutic agent whose usefulness is often negated by the development of drug resistance. There has been increasing support in the literature that altered expression of specific β-tubulin isotypes, in response to the development of resistance to antimitotic drugs such as Taxol, may be involved in this phenotype (Haber et al, 1995; Jaffrezou et al, 1995; Derry et al, 1997; Kavallaris et al, 1997; Ranganathan et al, 1996, 1998*a*, 1998*b*). Our laboratory recently has described increased expression of two brain-specific β-tubulin isotypes, Hβ4 (class III) and H5β (class IVa), in response to Taxol resistance in both human epithelial ovarian carcinoma and human lung cancer cell lines (Kavallaris et al, 1997). Antisense ODNs were selected against the Hβ4 β-tubulin gene since the expression level of this isotype was barely detectable in the parental A549 cells and it was the second most abundant isotype in the Taxol-resistant A549-T24 cells, after the constitutively expressed HM40 (class I) isotype. We describe here, for the first time, that inhibition of expression by antisense ODN of the class III β-tubulin isotype sensitizes Taxol-resistant cells to Taxol, strongly suggesting that tubulin isotypes are likely to be important in the development of resistance to Taxol.

Phosphorothioate antisense oligonucleotides were selected for this study due to their resistance to degradation by cellular nucleases and their relatively long half-life in culture (Helene and Toulme, 1990; Wagner, 1994). Furthermore, antisense ODNs have previously been used to inhibit the expression of proteins associated with drug resistance (Alahari et al, 1996; Stewart et al, 1996). We demonstrated that two ODNs, AS1 and AS3, could decrease Hβ4 mRNA levels by 40–50%. While antisense ODNs are a valuable tool for the study of gene function, it is important to ensure that the observed effects are specific for the desired gene and that appropriate controls are included (Helene and Toulme, 1990; Wagner, 1994).

Since ODNs in some instances can be degraded in culture and cause non-specific effects (Wagner, 1994), a matched scrambled ODN, SCR 2, was utilized in our study which had the same length and a similar base composition as that of the AS1 ODN. Down-regulation of the expression of the class III β-tubulin gene, Hβ4, was specific to this isotype as no change was observed in the levels of the constitutively expressed HM40 gene, or any of the other β-tubulin isotypes. Following AS1 ODN treatment, a significant decrease in the staining intensity of the class III β-tubulin protein was observed. This effect was specific to the class III protein, as no change in the total α-tubulin staining was visible. Total α-tubulin levels are often coordinately regulated with total β-tubulin levels (Gonzalez-Garay and Cabral, 1995). Since the total α-tubulin levels were similar between the AS1 treatment and the two controls, the down-regulation of the class III β-tubulin did not appear to affect the total tubulin levels. Of significance is the observation that the decreased expression of the class III β-tubulin protein in the A549-T24 cells resulted in an increase in sensitivity to Taxol. Our study clearly demonstrates that AS1 ODN specifically targets the Hβ4 message and that this results in decreased levels of the class III protein with a concomitant increase in Taxol sensitivity.

Microtubules are dynamic structures that are in a state of dynamic instability, that is, continuously growing and shortening (Mitchison and Kirschner, 1984). These important cytoskeletal

 © 1999 Cancer Research Campaign proteins are critical for cell growth and division, and are the target of a number of important chemotherapeutic agents (Jordan and Wilson, 1998). It has been demonstrated that microtubule dynamics in vitro are influenced by the β-tubulin isotype composition (Panda et al, 1994). In addition, microtubules depleted of class III β-tubulin polymerize more readily in the presence of Taxol than microtubules that are not depleted (Lu and Luduena, 1993). These data suggest that cells expressing higher levels of the class III β-tubulin isotype may be less sensitive to the effects of Taxol than cells expressing lower levels of this isotype. Recently, Derry et al (1997), using purified β-tubulin isotypes, demonstrated that microtubules composed of the class III or class IV β-tubulin isotype were approximately sevenfold less sensitive to the effects of Taxol than microtubules assembled from unfractionated tubulin. This study strongly indicated that β-tubulin isotype composition influences the sensitivity of microtubule dynamics to bound Taxol. Consistent with this proposal, decreasing the levels of class III β-tubulin in Taxol-resistant cells increased their sensitivity to Taxol.

We have previously suggested that expression of specific β-tubulin isotypes could alter microtubule dynamics in a way that could reduce the effectiveness of Taxol (Kavallaris et al, 1997). In support of this, a recent study found a positive correlation between increasing resistance to Taxol and increasing expression of the class III β-tubulin isotype among Taxol-resistant human prostate carcinoma cells (Ranganathan et al, 1998*a*). These data are consistent with our findings in Taxol-resistant human lung cancer cells in which increased levels of the class III β-tubulin isotype corresponded to an increase in Taxol resistance (Kavallaris et al, 1997). Furthermore, two human brain carcinoma cell lines which intrinsically expressed high levels of the class III β-tubulin isotype were significantly more resistant to Taxol than one which had no detectable levels as determined by immunoblot analysis (Ranganathan et al, 1998*b*). All three brain carcinoma cell lines accumulated equal amounts of Taxol, eliminating decreased accumulation of drug as a possible mechanism for decreased sensitivity to Taxol.

Tubulin is the target of a number of chemotherapeutic agents and a broader understanding of the interaction of these drugs in the tubulin/microtubule system could lead to improved drug targeting. Our study provides the first direct evidence that class III β-tubulin isotype levels in a cell can modulate response to Taxol. This study also raises the potential for therapeutic use of antisense oligonucleotides to specific β-tubulin isotypes to modulate tumour response to Taxol and other drugs.

### ACKNOWLEDGEMENTS

This study was undertaken during the tenure of a Research Training Fellowship awarded by the International Agency for Research on Cancer (MK). CAB was supported in part by National Institute of General Medical Services Training Program in Pharmacological Sciences grant 5T32 GM07260. This research was supported by US Public Health Service grant CA39821 (SBH) and Cancer Core Support grant CA13330 (SBH).

### REFERENCES

Akhtar S and Juliano RL (1992) Cellular uptake and intracellular fate of antisense oligonucleotides. *Trends Cell Biol* **2**: 139–143

Alahari SK, Dean NM, Fisher MH, Delong R, Manoharan M, Tivel KL and Juliano RL (1996) Inhibition of expression of the multidrug resistance-associated P-glycoprotein by phosphorothioate antisense oligonucleotides. *Mol Pharmacol* **50**: 808–819

Banerjee A, Roach MC, Trcka P and Luduena RF (1990) Increased microtubule assembly in bovine brain tubulin lacking the type III isotype of β-tubulin. *J Biol Chem* **165**: 1794–1799

Derry WB, Wilson S, Khan IA, Luduena RF and Jordan MA (1997) Taxol differentially modulates the dynamics of microtubules assembled from unfractionated and purified β-tubulin isotypes. *Biochemistry* **36**: 3554–3562

Dumontet C, Duran GE, Steger KA, Beketic-Oreskovic L and Sikic BI (1996) Resistance mechanisms in human sarcoma mutants derived by single-step exposure to Paclitaxel (taxol). *Cancer Res* **56**: 1091–1097

Giannakakou P, Sackett DL, Kang YK, Zhan Z, Buters JT, Fojo T and Poruchynsky MS (1997) Paclitaxel-resistant human ovarian cancer cells have mutant beta-tubulins that exhibit impaired paclitaxel-driven polymerization. *J Biol Chem* **272**: 17118–17125

Gonzalez-Garay ML and Cabral F (1995) Overexpression of an epitope-tagged β-tubulin in chinese hamster ovary cells causes an increase in endogenous α-tubulin synthesis. *Cell Motil Cytoskel* **31**: 259–272

Haber M, Burkhart CA, Regl DL, Madafiglio J, Norris MD and Horwitz SB (1995) Altered expression of Mβ2, the class II β-tubulin isotype, in a murine J744.2 cell line with a high level of taxol resistance. *J Biol Chem* **270**: 31269–31275

Helene C and Toulme JJ (1990) Specific regulation of gene expression by antisense, sense and antigene nucleic acids. *Biochim Biophys Acta* **1049**: 99–125

Horwitz SB, Cohen D, Rao S, Ringel I, Shen HJ and Yang CP (1993) Taxol: mechanisms of action and resistance. *J Natl Cancer Inst Monogr* **15**: 55–61

Hyams JS and Lloyd CW (1994) *Microtubules*. Wiley-Liss: New York.

Jaffrezou J-P, Dumontet C, Derry WB, Duran G, Chen G, Tsuchiya E, Wilson L, Jordan MA and Sikic BL (1995) Novel mechanism of resistance to Paclitaxel (Taxol®) in human K562 leukemia cells by combined selection with PSC 833. *Oncol Res* **7**: 517–527

Jordan MA and Wilson L (1998) Microtubules and actin filaments: dynamic targets for cancer chemotherapy. *Curr Opin Cell Biol* **10**: 123–130

Kavallaris M, Kuo DY-S, Burkhart CA, Regl DL, Norris MD, Haber M and Horwitz SB (1997) Taxol-resistant epithelial ovarian tumors are associated with altered expression of specific β-tubulin isotypes. *J Clin Invest* **100**: 1282–1293

Lewis SA and Cowan NJ (1990) Tubulin genes: structure, expression, and regulation. In: *Microtubule Proteins*, Avila J (ed), pp. 37–66. CRC Press, Inc.: Boca Raton, Florida.

Lu Q and Luduena RF (1993) Removal of $\beta_{III}$ isotype enhances taxol induced microtubule assembly. *Cell Struct Funct* **18**: 173–182

Luduena RF (1998) Multiple forms of tubulin: different gene products and covalent modifications. *Int Rev Cytol* **178**: 207–274

Mitchison TJ and Kirschner MW (1984) Dynamic instability of microtubule growth. *Nature* **312**: 237–242

Panda D, Miller HP, Banerjee A, Luduena RF and Wilson L (1994) Microtubule dynamics in vitro are regulated by the tubulin isotype composition. *Proc Natl Acad Sci* **91**: 11358–11362

Ranganathan S, Dexter DW, Benetatos CA, Chapman AE, Tew KD & Hudes GR (1996) Increase of $\beta_{III}$- and $\beta_{IVa}$-tubulin in human prostate carcinoma cells as a result of estramustine resistance. *Cancer Res* **56**: 2584–2589

Ranganathan S, Benetatos CA, Colarusso PJ, Dexter DW and Hudes GR (1998*a*) Altered beta-tubulin isotype expression in paclitaxel-resistant human prostate carcinoma cells. *Br J Cancer* **77**: 562–566

Ranganathan S, Dexter DW, Benetatos CA & Hudes GR (1998*b*) Cloning and sequencing of human betaIII-tubulin cDNA: induction of betaIII isotype in human prostate carcinoma cells by acute exposure to antimicrotubule agents. *Biochem Biophys Acta* **1395**: 237–245

Rao S, Orr GA, Chaudhary AG, Kingston DGI and Horwitz SB (1995) Characterization of the taxol binding site on the microtubule. *J Biol Chem* **270**: 20235–20238

Stewart AJ, Canitrot Y, Baracchini E, Dean NM, Deeley RG and Cole SPC (1996) Reduction of expression of the multidrug resistance protein (MRP) in human tumor cells by antisense phosphorothioate oligonucleotides. *Biochem Pharmacol* **51**: 461–469

Sullivan KF and Cleveland DW (1986) Identification of conserved isotype-defining variable region sequences for four vertebrate β tubulin polypeptide classes. *Proc Natl Acad Sci USA* **83**: 4327–4331

Wagner RW (1994) Gene inhibition using antisense oligonucleotides. *Nature* **372**: 333–335

*© 1999 Cancer Research Campaign*
